# Decontamination of SARS‐CoV‐2 contaminated N95 filtering facepiece respirators using artificial sun lamps

**DOI:** 10.1111/jam.15106

**Published:** 2021-05-06

**Authors:** D.C. Glasbrenner, Y.W. Choi, A.W. Richardson, E.W. Edwards, M.J. Mladineo, M. Sunderman, P.H. Keyes, J. Boyce, J.K. Middleton, M.W. Howard

**Affiliations:** ^1^ Battelle Memorial Institute Columbus OH USA

**Keywords:** disinfection, environmental health, non‐thermal processes, ultraviolet applications, viruses

## Abstract

**Aims:**

Assess the feasibility of using light from artificial sun lamps to decontaminate N95 filtering facepiece respirators (FFRs) contaminated with SARS‐CoV‐2.

**Methods and Results:**

FFR coupons or whole FFRs contaminated with 5 log_10_ TCID_50_ (target concentration) SARS‐CoV‐2 in culture media, simulated saliva, or simulated lung fluid were dried for 1–2 h, then exposed to light from tanning and horticulture lamps to assess decontamination. Exposed coupons and whole FFRs showed SARS‐CoV‐2 inactivation for all matrices tested. Furthermore, FFRs still met performance specifications after five decontamination cycles.

**Conclusions:**

It is feasible that artificial sunlight from these sun lamps can be used to decontaminate FFRs provided the UV dose is sufficient and the light is unobstructed. Furthermore, decontamination can be performed up to five times without degrading FFR performance.

**Significance and Impact of the Study:**

This research shows a proof of principle that artificial sun lamps may be an option to decontaminate SARS‐CoV‐2 on N95 FFRs. UV doses required for inactivation to levels below detection ranged from 4 to 37·8 J cm^−2^ depending on the light source, virus matrix and FFR type.

## Introduction

N95 filtering facepiece respirators (FFRs) are a critical resource used to reduce the possibility of infection for healthcare workers, first responders and other frontline workers (Iannone *et al*. 2020). The potential for a pandemic to cause shortages of N95 FFRs has been recognized for over a decade (Anon. 2006). Since that time, researchers have examined various methods to decontaminate FFRs should critical shortages occur during a public health crisis. Methods examined include physical and chemical treatments with the objective of inactivating pathogens or surrogate organisms without impacting FFR performance (Viscusi *et al*. 2009; Bergman *et al*. 2010; Heimbuch *et al*. 2011; Viscusi *et al*. 2011; Lore *et al*. 2012; Lin *et al*. 2018; Mills *et al*. 2018; Aboubakr *et al*. 2020; Cadnum *et al*. 2020; Fischer *et al*. 2020; Pauley *et al*. 2020).

Exposure to ultraviolet germicidal irradiation (UVGI) is one of the methods identified as promising by the Centers for Disease Control (CDC), National Institute for Occupational Safety and Health (NIOSH) (Anon. 2020a, 2020b). UVGI has been shown to inactivate influenza virus (Heimbuch *et al*. 2011; Lore *et al*. 2012; Mills *et al*. 2018), methicillin‐resistant *Staphylococcus aureus* (MRSA), MS2 phage (Cadnum *et al*. 2020), Phi6 phage (Cadnum *et al*. 2020) and *Bacillus subtilis* spores (Lin *et al*. 2018) when present on various FFR models. In addition, it has been shown separately that FFRs still meet aerosol collection efficiency and breathing resistance requirements after UVGI exposure (Viscusi *et al*. 2009, 2011). However, the potential for UVGI to reduce polymer strength on some FFR models making them more vulnerable to damage from physical stresses has been noted (Lindsley *et al*. 2015). As of this writing, the U.S. Food and Drug Administration (FDA) has issued one Emergency Use Authorization (EUA) for a system using UVGI for FFR decontamination for use by healthcare professionals (Hinton 2020). Despite efficacy shown in previous research and the issuance of EUAs, it should be remembered that decontamination for reuse is an option of last resort and not recommended by NIOSH because this is not consistent with the approved use of the device.

Separate from the UVGI work, it has been shown that artificial sunlight inactivates SARS‐CoV‐2 when present on non‐porous surfaces or in aerosols (Ratnesar‐Shumate *et al*. 2020; Schuit *et al*. 2020). Artificial sunlight differs from UVGI in that it does not contain light in the UV‐C wavelength range of 100–280 nm but does contain ultraviolet light in the UV‐B (280–315 nm) and UV‐A (315–400 nm) wavelengths. SARS‐CoV‐2 inactivation by artificial sunlight opens the possibility that artificial sun lamps used in tanning and horticulture consumer products could be used for FFR decontamination. These artificial sun lamps are available through retail stores, online shopping, or could be repurposed in a fee for use fashion in the case of tanning beds which are present in tanning salons across the country. As a result, they could be used to meet the needs of persons not covered by the EUA should replacement FFRs become unavailable and the needs for respiratory protection remain. The feasibility of using artificial sunlight from these sun lamps was first demonstrated using a surrogate alpha‐coronavirus transmissible gastroenteritis virus (TGEV) inoculated onto coupons of FFRs which was tested in our Biosafety Level‐2 (BSL‐2) laboratory to assess feasibility and perform troubleshooting before working with SARS‐CoV‐2 in the Biosafety Level‐3 (BSL‐3) laboratory. Efficacy against SARS‐CoV‐2 is then confirmed with virus in three different matrices inoculated onto coupons or whole FFRs. Finally, it is shown that exposure to artificial sunlight does not degrade FFR performance following up to five decontamination cycles.

## Materials and methods

### Cells and virus

Swine testicular cells (ST cells, ATCC Cat. No. CRL‐1746) were used to propagate and perform virus infectivity assays of TGEV (Purdue Strain, ATCC VR‐763). ST cells were incubated at 37°C with 5% carbon dioxide (CO_2_) in complete Dulbecco’s Modified Essential Medium (DMEM, Gibco, cat. no. 10566016) supplemented with 10% fetal bovine serum (FBS, Omega Scientific, Cat. No. FB‐02) and 1% Penicillin‐Streptomycin (PS, Gibco Cat. No. 15140122). ST cells never exceeded the fifth passage from the purchased stock. Monolayers of ST cells at 80–90% confluency were washed with completed DMEM except the amount of FBS is reduced to 2% as this improves virus production based upon data from our laboratory. After conditioning cells for 3 h, media was removed, and virus in supernatant from a laboratory prepared working stock was added for a multiplicity of infection (MOI) of 0·01 and adsorbed for 1 h at 37°C and 5% CO_2_ with shaking. Following adsorption, the 2% FBS DMEM with 1% PS was added and flasks were incubated for 48 h at 37°C and 5% CO_2_. Forty‐eight hours post‐infection, flasks were frozen at −80°C in an ultra‐low freezer (Forma Scientific 8520, Waltham, MA) for at least one hour, thawed at ambient temperature in the biosafety cabinet in the dark, and viral lysate was collected after centrifugal clarification (800 ***g***, 5 min). Clarified viral lysate was aliquoted and frozen and stored at <−60°C in single‐use vials for testing.

Vero (African green monkey kidney) clone E6 cells (BEI Resources product No. NR‐596, Manassas, VA) were used to propagate SARS‐CoV‐2 strain USA‐WA1/2020 (BEI Resources, Manassas, VA) and perform virus infectivity assays. Vero clone E6 cells were incubated at 37°C with 5% CO_2_ in complete Eagles Minimal Essential Medium (EMEM, Corning, NY, Cat. No. 10‐010‐CM) supplemented with 10% FBS (Gibco Cat. No. 10082147) and PS (Gibco Cat. No. 15140122). For infection, culture media was removed from the roller bottle and 2 ml of laboratory prepared virus stock was added (MOI of 0·001) along with 5 ml of EMEM supplemented with 5% FBS and PS and allowed to absorb for 1‐h at 37°C. After absorption, 25 ml of complete EMEM (5% FBS, PS) were added and culture continued for 36–48 h at 37°C. Virus was harvested when cytopathic effect (CPE) was apparent throughout the flask and any remaining cells were removed by scraping. Infected cells were further disrupted by vortexing (Fisher Scientific, Waltham, MA, Cat. No. 02215452) for 2 min at maximum speed and ambient temperature with sterile glass beads (Sigma‐Aldrich, St. Louis, MO, Cat. No. CLS72685) at a ratio of 1 : 7 beads to cells, which provides greater yield than freeze‐thaw in our experience, and then centrifuged at 800 ***g*** for 5 min at 4°C to remove any remaining cellular debris. The resulting suspension was aliquoted and frozen at −80°C in single‐use vials for testing. All work done with SARS‐CoV‐2 was done in a Biosafety Level 3 (BSL‐3) laboratory.

Virus infectivity was quantified by end point dilution assay to determine the median tissue culture infectious dose (TCID_50_). For TGEV, 80–90% confluent monolayers of ST cells were conditioned with 2% FBS containing DMEM culture medium for 2–4 h at 37°C and 5% CO_2_. Ten‐fold serial dilutions were plated onto conditioned ST cells, with wells scored for CPE based upon researcher observation 48 h post‐infection as determined by the virus growth rate. The limit of detection for the TGEV infectivity assay was 10 TCID_50_ as determined by CPE being observed in the lowest dilution. For SARS‐CoV‐2, 10‐fold serial dilutions in completed EMEM (5% FBS, PS) were plated onto 80–90% confluent Vero E6 monolayers, with wells scored for CPE 72 h post‐infection as determined by the virus growth rate. Quantification of the titre was determined via the Spearman–Karber method (Hamilton *et al*. 1977). The limit of detection for the SARS‐CoV‐2 TCID_50_ assay was 13·1 TCID_50_ (1·12 log_10_ TCID_50_) as determined by CPE being observed in the lowest dilution.

### Filtering facepiece respirators

Four types of N95 FFRs were selected for this study. These FFRs were selected based upon their common use in the field, presence of unique features, and availability given that FFRs were in extremely short supply during this research effort. The FFRs used were 3M Models 1860, 8210 and 8511 (St. Paul, MN) and Northern Safety (NS) Model 7210 (Utica, NY). The 3M 1860 and 3M 8210 are commonly used by healthcare professionals, first responders and security professionals who interact with the public. The 3M 8511 is unique in this study since it has an exhalation valve, and the NS 7210 is from a different manufacturer to help understand whether the results generated may be applicable to multiple manufacturers.

### *Artificial* *Sun Lamps*

Three candidates for artificial sun lamps were identified. The Sperti Fiji Sun Home Tanning Lamp (Model FIJI101911071911, KBD, Inc. Las Vegas, NV) is an in‐home tanning product. The Flower Power bulb (Model FR40T12‐H0, Solacure, Browns Summit, NC) was purchased along with the manufacturer recommended ballasts and is available to gardening hobbyists. These items were readily available through Amazon (www.amazon.com). Finally, an Ergoline Affinity 600 Turbo Power Tanning Bed Model JK 92/46‐3 (AC) (Suntan Supply, Avon, OH) was purchased for use in the study and represents the abundance of tanning facilities across the country. The Ergoline Affinity 600 Turbo Power Tanning Bed was provided with Wolff GoldenBronze^TM^ Plus bulbs (Cat. No. GBP‐T12‐71‐160WR BI‐PIN). Note, only a single unit of each of these devices was tested and hence the potential variability between different devices has not been assessed in this study.

Spectral characterization was conducted using an Ocean Optics USB4000 fiber optic spectrometer (Largo, FL). The input to the spectrometer is a subminiature version A (SMA) adapter that was coupled to a collimating lens to gather light from the various sources at an appropriate standoff distance. Spectral intensities were collected from 200 to 1000 nm. Data were collected with 3648 data points over the 200–1000 nm range with 1000 scans combined into an average spectrum. Exposure time was selected to maximize the signal without exceeding the limits of the detector and ranged from 3·8 to 10·0 ms. After collection, data were processed to provide the relative intensity on a scale of 0·0–1·0 with 1·0 corresponding to the maximum number of counts observed in the average scans.

Light spectra and intensities were measured using a Thorlabs (Newton, NJ) PM100D Light Power Meter and a Thorlabs S120VC Photodiode having a range from 200 to 1100 nm. The Photodiode was fit with either Semrock filter (Lake Forest, IL) FF02 320/40‐25 for 300 to 340 nm, or Semrock filter FF01 378/52/25 for 352–404 nm. Power was measured with each filter serially. Light collected from 300 to 340 nm was power corrected at 325 nm. The power from this reading was adjusted to account for the gap in the two filter sets from 340 to 350 nm. Light collected from 352 to 404 nm was power corrected at 375 nm. Measured values were increased to account for Semrock published losses in the filters. For measurements that required placing a sample in a polyethylene bag the dose delivered to the sample was taken at 90% of the nominal measurement value to account for transmission losses through the bag. UV dose measurements are reported as the optical energy per unit area (J cm^−2^) delivered to the sample from 300 to 400 nm. The Sperti Fiji Sun lamp and the Flower Power bulb were mounted in a fixture that maintained the position of the lamps from the sample at 11·5 inches and allowed mapping of the intensity on the test surface in a 1‐inch by 1‐inch grid which was used to guide sample placement. To place the tanning bed into the BSL‐3 laboratory it was necessary to remove the canopy and some of the decorative pieces. Thus, all testing was done without the canopy and the controls were placed on a long tether to allow operation of the tanning bed without exposing the operators to UV irradiation. Due to the size of the test area and relevant size of the samples a 2‐inch by 2‐inch grid was used for the tanning bed and samples were placed directly on top of tanning bed surface where a person lies.

### Simulated saliva and simulated lung fluid

Simulated saliva was prepared in 500 ml batches as described by Biryukov *et al*. (2020) with the salts being weighed and dissolved into double deionized water according to their provided recipe (see Supporting Information) along with porcine mucin (Lee Biosolutions, Maryland Heights, MO, Cat. No. 435‐11) at a final concentration of 3 g l^−1^ and the pH was adjusted to 7·0. Simulated saliva was stored at 4°C until use and any unused portion was discarded after 1 week and a new preparation was made. Simulated lung fluid was prepared based upon the work of Hassoun *et al*. (2018) and Kumar *et al*. (2017) and modified to use Hanks’ Balanced Salt Solution (HBSS) as the diluent for the protein and antioxidant components (Bicer 2014). DPPG, DPPC, and cholesterol solutions were prepared in chloroform, mixed in the correct proportions and the chloroform allowed to evaporate. Other components were combined in HBSS to their final concentrations (see Supporting Information), added to the lipid/cholesterol mixture and then sonicated (Sonics, Newton, CT, Vibra Cell Model VC 505 with Probe model CV336) for 10 min at an amplitude of 20% with a 30 s on, 30 s off cycle.

Virus was concentrated and resuspended in simulated saliva or simulated lung fluid using a centrifugal concentrator (Spin‐X UF Concentrator, Corning Cat. No. CLS431491, Corning, NY) with a 100 kiloDalton (kDa) molecular weight cutoff that retained the SARS‐CoV‐2 but allowed the complete cell culture media components to be removed. The retentate virus was resuspended (i.e. *quantum satis*; Q.S.) with either the simulated saliva or simulated lung fluid prior to spiking representative FFR coupons or whole masks. Virus concentrations in simulated saliva were approximately 4·4 log TCID_50_ and 5·8 log TCID_50_ in simulated lung fluid.

### *Virus* *inactivation assays*

Inactivation was performed with representative coupons from the FFRs or with whole FFRs as indicated. Coupons were 2 × 2 cm and multiple coupons were cut from each FFR. Coupons were inoculated with 100 *μ*l of either TGEV or SARS‐CoV‐2 stock by placing droplets (~10 *μ*l) onto the coupon. Droplets were allowed to dry on the coupons by evaporation (1–2 h) in a sealed container on a bed of Drierite desiccant (WA Hammond Drierite Co., LTD, Xenia, OH) at ambient temperature. After drying, coupons were placed into small square polyethylene‐low density polymer plastic bags (cut from Ziploc bags) and sealed to simulate a reasonable practice for users to prevent transfer of virus from contaminated FFRs to other surfaces. Positive control coupons and whole FFRs were tested in triplicate and used to determine the starting titre of virus inoculated on the test samples. Treated samples were tested in triplicate using a single virus preparation and subjected to UV light for preset times with the time and sample's location on the test surface being recorded. A negative control was included with each sample (positive control or treated) to confirm that material from the FFR was not affecting the viability assay. The tanning bed controls limited single exposures to 12 min, thus longer exposures for higher doses were achieved by running the 12‐min cycle multiple times until the desired accumulative dose was achieved. Virus was extracted from each coupon by placing it in 10 ml of complete DMEM that included 2% FBS and then agitating for 15 min at 200 rotations per minute (RPM) on a platform orbital shaker. The solution was then removed from the tube and concentrated down to 2 ml using a centrifugal concentrator (Spin‐X UF Concentrator, Corning Cat. No. CLS431491) with a 100 kDa cutoff. The 2‐ml concentrated samples were then filter‐sterilized (Thermo‐Fisher Cat. No. 720‐1320, Waltham, MA) through a low‐binding, 0·2‐*μ*m filter before being assayed for infectious virus by the TCID_50_ assay.

When whole FFRs were used, four locations for virus inoculation of 2 cm × 2 cm square were marked so that efficacy of inactivation could be assessed across the curved shape of the FFR. Virus was inoculated within each of the marked locations and dried as described for coupon samples. Full FFRs were then sealed in plastic bags and exposed to artificial sunlight. Once the appropriate exposure was completed, these mapped locations were then excised and processed in the same manner as the coupons. The results from each of the locations were treated as replicates for the sample with mean and standard deviation values reported.

The UV dose received by the coupons or whole FFRs was calculated using the exposure time and irradiance measured at each location. Replicate samples were placed in adjacent locations, harvested at the same time, and the UV dose per coupon was considered the average of the dose received within each location. Times, measured UV doses, and inactivation data are tabulated in the Supporting Information.

### Performance degradation testing

Assessment of performance degradation included determination of the initial collection efficiency, inhalation resistance, change in strap elasticity and visual inspection of whole FFRs. Collection efficiency and breathing resistance were determined using the 8130A automated filter tester (TSI Inc., Shoreview, MN) in a manner consistent with procedure number TEB‐APR‐STP‐0059 of the NIOSH (Anon. 2019), except that only initial collection efficiency was measured, instead of measuring it after loading 200 mg of salt onto the FFR and the FFRs were not subjected to pre‐conditioning at 38°C and 85% relative humidity prior to testing. This was designed so that any changes in performance could be attributed solely to the UV exposure. Whole FFRs were glued onto the test plate using hot glue to ensure a seal along the edge and then placed into the tester. Whole FFRs were challenged with a salt aerosol and the tester reported the collection efficiency and inhalation resistance at 85 l min^−1^. Strap elasticity was tested by performing a three‐step process where a 10‐inch section of the strap was stretched three times to 200, 150 and then 200% of its length at a rate of 1 cm s^−1^. On the final stretch the maximum stress and maximum load on the strap was recorded for comparison. Visual inspection examined the integrity of the filtration media, strap attachments, nose pads and exhalation valve (if present).

## Results

### Characterization of the artificial sun lamps

**Figure 1:**
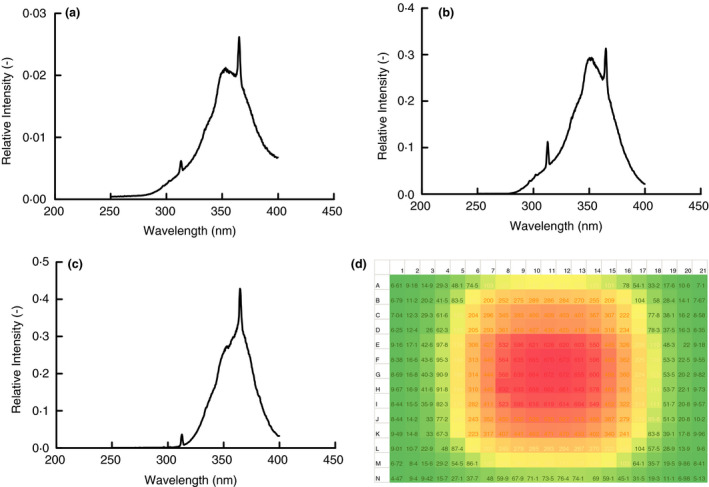
Light spectra for the artificial sun lamps examined with focus on the 300–400 nm range of wavelengths and corrected at 325 nm. Relative intensities at different wavelengths were measured using a ThorLabs optical power meter with appropriate Semrock filters. Presented is a single measurement of the spectrum for each lamp.

Prior to testing, the light spectrum of each of the artificial sun lamps was measured. Figure 1 shows the results of those measurements with focus on the UV range of the light (200–400 nm). Each lamp showed a broad spectrum of wavelengths from 300 to 400 nm with small but distinct peaks at 313 and 365 nm. These lamps differed in intensity with the tanning bed being the strongest at 11 mW cm^−2^, the Sperti Fiji Sun Lamp being the next strongest with an output of 0·72 mW cm^−2^ and the Flower Power bulbs (two included in the test fixture) being the least intense with an output of 0·37 mW cm^−2^. Also shown in Fig. 1 is the irradiance map for the Sperti Fiji Sun lamp that shows the dose by position on the test surface for a given time. Similar maps were made for the test surface in all tests conducted so that the UV dose for each sample could be calculated from their position and duration of exposure.

### Inactivation of TGEV on FFR coupons using simulated sun lamps

**Figure 2:**
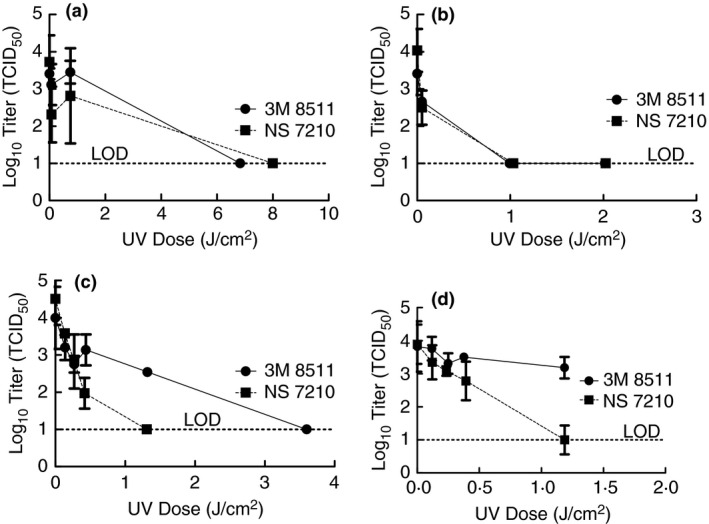
Inactivation of TGEV using artificial sunlight from the Sperti Fiji Sun lamp for 0·7, 7, and 77 min (a), Flower Power bulb for 3·5, 35, and 70 min (b), and tanning bed for 0·22, 0·43, 0·67, 2, and 5 min (c) with demonstration of TGEV inactivation on whole FFRs in the tanning bed for 0·22, 0·43, 0·67, and 2 min (d). Displayed is the mean and standard deviation of the log10 TCID_50_. Limit of detection for the experiment is 10 TCID_50_. (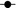) 3M 8511; (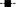) NS 7210.

Prior to conducting work in the BSL‐3 laboratory, it was desired to test the efficacy of artificial sunlight from these sun lamp options for inactivation using a surrogate virus. TGEV was selected as the surrogate because it is a coronavirus and was available in our laboratory. Due to the COVID‐19 pandemic and limited FFR supplies, virus inactivation tests were performed using coupons cut from whole FFRs (i.e. N95s) to conserve FFR quantities. Furthermore, only the 3M 8511 and NS 7210 models were used in surrogate tests because quantities of the 3M 1860 and 3M 8210 were very limited and reserved for testing with SARS‐CoV‐2. For the surrogate tests, TGEV in culture media was inoculated onto FFR coupons and exposed to artificial sunlight from each sun lamp (see Fig. 2a–c). In each case, there was a dose at which TGEV was inactivated to levels below detection.

Having demonstrated that inactivation of TGEV on FFR coupons was possible with artificial sunlight from these sun lamps, it was desired to ascertain whether the shape of the FFRs would impact the feasibility of this method to be used for FFR decontamination purposes. For this test, whole FFRs were inoculated with TGEV in culture media at four locations on the mask, placed inside a Ziploc sandwich bag, and then exposed to artificial sunlight on the tanning bed. In each case, virus inactivation occurred, but for the 3M 8511 the extent of inactivation was significantly lower for the same dose suggesting higher UV doses will be necessary when decontaminating whole 3M 8511 FFRs (Fig. 2d). This fact was taken into consideration with subsequent tests using SARS‐CoV‐2 but these results were not further refined for TGEV as generating results for SARS‐CoV‐2 was a higher priority with our limited FFR quantities.

### Inactivation of SARS‐CoV‐2 on FFRs using simulated sun lamps

**Figure 3:**
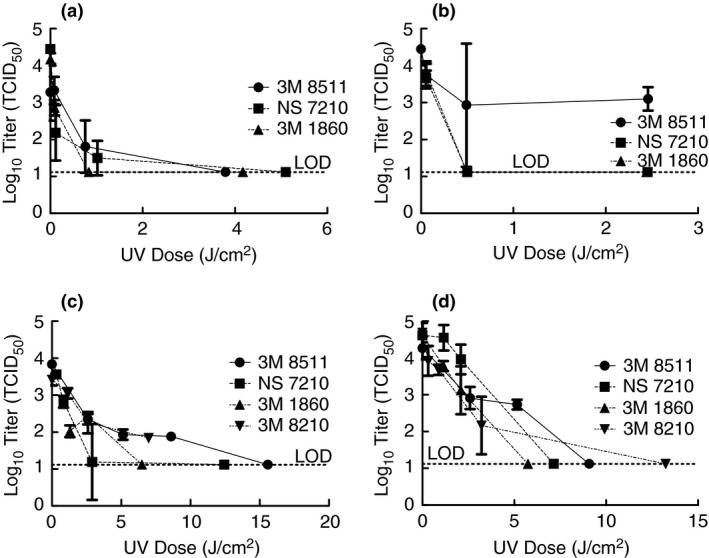
Inactivation of SARS‐CoV‐2 using artificial sunlight from the Sperti Fiji Sun lamp (a), Flower Power bulb (b), and tanning bed (c) with demonstration of SARS‐CoV‐2 inactivation on whole FFRs in the tanning bed (d). Displayed is the mean and standard deviation of the log10 TCID_50_. Limit of detection for the experiment is 13·1 TCID_50_ (1·12 log10 TCID_50_). (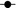) 3M 8511; (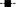) NS 7210; (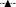) 3M 1860; (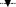) 3M 8210.

After demonstrating that artificial sunlight from commercial sun lamps was sufficient to inactivate TGEV, it was desired to determine if the same would be true for SARS‐CoV‐2. Test apparatuses were moved to the BSL‐3 laboratory, including the tanning bed, and the experiments were performed with SARS‐CoV‐2. Coupons from FFRs were inoculated with SARS‐CoV‐2 in culture media, exposed to artificial sunlight for 1, 9 and 45 min (for all FFR models), and the amount of viable virus remaining was determined (Fig. 3a–c). Note, the quantities of 3M 8210 FFRs were very limited and more could not be acquired so they are only included in the tanning bed tests. For the three FFR models tested with the Sperti Fiji Sun lamp (Fig. 3a), significant inactivation occurred by a dose of approximately 1 J cm^−2^ and to levels below detection by approximately 4 J cm^−2^. In tests with the Flower Power bulb, exposure times were 1, 9 and 45 min and SARS‐CoV‐2 was inactivated to levels below detection on the 3M 1860 and NS 7210 FFRs by approximately 0·5 J cm^−2^, but titre remained on the 3M 8511 FFR coupons at values up to 2·4 J cm^−2^. It is thought that this is partially due to the filter media on the 3M 8511 being more absorbent and hence the virus inoculum likely penetrated further into the media making it more difficult for the light to reach the virus contained therein. In addition, the large error bar on the 3M 8511 at a dose of 0·5 J cm^−2^ is due to one of the replicates having a lower value, however, no issues with the test could be identified and hence the replicate was included. In addition, points throughout the tests tend to have larger error bars as they approach the limit of detection as some replicates will have a measurable titre while others do not which caused the standard deviation to be higher. Tests with the Affinity 600 tanning bed were conducted with exposure times differing by the FFR type and varied from as short as 0·5 min to as long as 30 min. The 3M 8511 FFR coupons required higher UV doses to achieve inactivation below detection at approximately 16 J cm^−2^ (30 min). On the tanning bed, the 3M 8210 FFR coupons also required higher doses with 1·84 log_10_ TCID_50_ remaining after a dose of 7 J cm^−2^ (12 min). Unfortunately, additional coupons of the 3M 8210 FFR were not available to test higher doses, but higher doses were tested with whole masks (see Fig. 3d and discussion below). Inactivation below detection in the tanning bed occurred at doses of 3 J cm^−2^ (5 min) for the NS7210 FFR coupons and 6·5 J cm^−2^ (10 min) for the 3M 1860 FFR coupons.

Findings with the FFR coupons were confirmed using whole FFRs for exposure to artificial sunlight from the tanning bed with the exposure times being specific to the FFR type and ranging from 0·5 to 60 min (Fig. 3d). SARS‐CoV‐2 in culture media was inoculated onto the FFR in four locations and then exposed to artificial sunlight on the tanning bed. Inactivation below detection occurred at approximately 6 J cm^−2^ (10 min) for the 3 M 1860 FFR and 7 J cm^−2^ (12 min) for the NS 7210 FFRs. Higher doses were necessary for inactivation below detection for the 3M 8210 and the 3M 8511. However, at doses of at least 15 J cm^−2^ (30 min) there was inactivation below detection for all masks tested.

**Figure 4:**
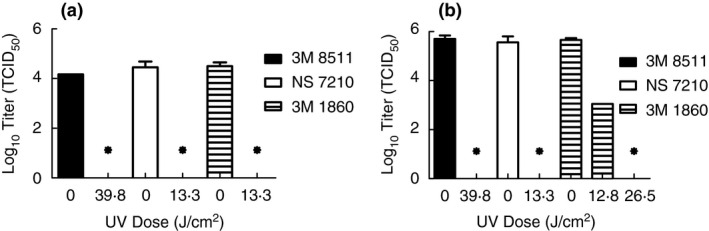
Survival of SARS‐CoV‐2 in simulated saliva (a) and simulated lung fluid (b) using artificial sunlight from a tanning bed. Displayed is the mean and standard deviation of the log10 TCID_50_. Limit of detection for the experiment is 13·1 TCID_50_ (1·12 log10 TCID_50_) and is indicated by an asterisk. (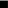) 3M 8511; (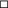) NS 7210; (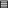) 3M 1860.

Finally, inactivation testing was performed with SARS‐CoV‐2 in simulated saliva and simulated lung fluid to evaluate whether or not exposure to artificial sunlight from the tanning bed would still cause inactivation below detection. Both solutions were selected as they represent possible matrices that could surround exhaled virus from an infected person. The different composition of these two solutions could impact decontamination and hence it was desired to consider both in assessing the feasibility of decontamination by artificial sun lamps. Tests were performed with coupons from the 3M 1860, 3M 8511 and NS 7210 FFRs (3M 8210 FFRs were not available) and end point doses were used to verify inactivation (Fig. 4). Inactivation below levels of detection were achieved for SARS‐CoV‐2 in simulated saliva at a dose of 13·3 J cm^−2^ (20 min) for the 3M 1860 and NS 7210 FFRs and a dose of 37·8 J cm^−2^ (60 min) for the 3M 8511. For simulated lung fluid, inactivation to levels below detection were observed at the same UV doses for the NS 7210 and 3M 8511 FFRs, but a dose of 26·5 J cm^−2^ (40 min) was needed for the 3M 1860 FFR. Still, UV doses at which artificial sunlight from the tanning bed were sufficient to inactivate SARS‐CoV‐2 to levels below detection were identified for all matrices and FFRs tested, and it was assessed that a single decontamination dose of 37·8 J cm^−2^ (60 min) would be the conservative assumption for decontamination in the tanning bed. Note, it may be possible to refine this dose level further, but additional FFR quantities were not available to conduct this testing.

Note, inoculation by droplets was chosen because it was simpler to apply a known quantity of virus to the coupon and conserve virus quantities. This inoculation method is different than filtering of aerosols as would be experienced during use where the aerosol may already be dry prior to impacting the FFR and may also penetrate further into the filter media as part of the filtration process. However, surface inoculation was deemed sufficient to demonstrate the feasibility of this approach.

### Assessment of FFR performance following decontamination with artificial sunlight

For a decontamination method to be acceptable, it must reduce the amount of viable virus on the FFR while not impacting the FFR’s performance. Having determined that artificial sunlight can inactivate SARS‐CoV‐2 in three different matrices and on up to four different FFRs, it was necessary to assess whether exposure to artificial sunlight would degrade FFR performance. It was decided to assess the impact of artificial sunlight from the tanning bed on FFR performance using the conservative dose of 37·8 J cm^−2^ as the requirement for a single decontamination cycle. Whole FFRs were subjected to decontamination with artificial sunlight for one or five decontamination cycles and then tested for performance (Table 1).

**Table 1:** Summary of performance testing results for FFRs exposed to decontamination with artificial sunlight in a tanning bed. Provided are the average measurements and their standard deviation

FFR model	UV dose* (J cm^−2^)	Collection efficiency^†^ (%)*	Breathing resistance^‡^ (mm H_2_O)^†^	Strap stress @ 200% Strain (MPa)	Strap load @ 200% Strain (N)	Visual inspection
3M 1860	0	99·7 ± 0·1	8·4 ± 0·6	1·21 ± 0·02	5·7 ± 0·07	N/A
	37·8	99·7 ± 0·06	7·2 ± 0·2	ND	ND	NVD
	189	99·7 ± 0·03	8·15 ± 1	1·26 ± 0·06	5·9 ± 0·3	NVD
3M 8210	0	99·7 ± 0·4	7·0 ± 0·5	1·44 ± 0·09	5·8 ± 0·1	N/A
	37·8	99·6 ± 0·2	6·3 ± 0·3	ND	ND	NVD
3M 8511	0	98·9 ± 0·8	6·2 ± 0·4	1·11 ± 0·1	6·0 ± 0·5	N/A
	37·8	98·8 ± 0·5	6·2 ± 0·3	ND	ND	NVD
	189	98·8 ± 0·6	6·2 ± 0·3	1·13 ± 0·08	5·6 ± 0·4	NVD
NS 7210	0	99·5 ± 0·2	9·1 ± 0·3	1·67 ± 0·05	4·9 ± 0·02	N/A
	37·8	99·4 ± 0·1	9·2 ± 0·7	ND	ND	NVD
	189	99·5 ± 0·1	8·3 ± 0·7	1·99 ± 0·1	5·1 ± 0·01	NVD

N/A = not applicable; ND = not determined; NVD = no visual defects.

* UV doses measure over 300–400 nm. Dose levels correlate to 1 h (37·8 J cm^−2^) and 5 h (189 J cm^−2^) of exposure to the tanning bed.

^†^ Requirement for N95 FFRs is >95%.

‡ Requirement for N95 FFRs is <32 mmH_2_O.

All FFRs tested met the requirements for collection efficiency and breathing resistance after one decontamination cycle (Anon. 2019). The three FFRs tested for five decontamination cycles also met the requirements for collection efficiency and breathing resistance. The 3M 8210 was not tested for five decontamination cycles because additional samples could not be acquired. In addition, the impact of decontamination by artificial sunlight on strap elasticity was measured. Only the change in stress for the NS 7210 sample proved to be statistically significant by one‐way ANOVA with *P* = 0·05 with a 19% change. All other changes were not statistically significant. Strap elasticity was measured as an indicator of fit and these results suggest that fit would not be impacted due to a change in strap elasticity by this decontamination method. In addition, exposure to artificial sun light from the tanning bed did not cause any damage to the FFRs that could be identified upon visual inspection. Still, these results are not a replacement for fit testing which can be impacted by changes in mask shape that are not possible to assess via strap elasticity or visual inspection. Thus, additional experiments including fit testing are needed to further determine if this method is suitable for decontamination for reuse.

## Discussion

This study has confirmed that artificial sunlight generated by tanning bed and horticulture artificial sun lamps can inactivate SARS‐CoV‐2 on FFRs without negatively impacting FFR performance. These findings are consistent with other research that has shown that SARS‐CoV‐2 on surfaces and in aerosols is inactivated when exposed to artificial sunlight (Ratnesar‐Shumate *et al*. 2020; Schuit *et al*. 2020). Because tanning and horticulture products are widely available, they represent an accessible and more affordable option for FFR decontamination for persons who desire respiratory protection, but do not have access to the options available through the FDA EUAs.

Feasibility testing with TGEV proved to be a reasonable indicator of how UV light from artificial sun lamps will inactivate SARS‐CoV‐2. Inactivation to levels below detection were observed above 2 J cm^−2^ for coupons of the NS 7210 mask for both TGEV and SARS‐CoV‐2 while higher doses were needed to achieve inactivation to levels below detection with the 3M 8511 mask. However, a more stringent comparison is not possible because FFRs were not available to allow additional UV doses to be tested with TGEV and SARS‐CoV‐2 so that the inactivation kinetics can be determined with greater resolution.

For SARS‐CoV‐2, differences in the UV dose required to get inactivation below detection were noticeably higher for the 3M 8511 FFR. It was observed that the inoculum was absorbed into the filter media for this mask which likely contributed to the need for a higher dose. Conversely, the hydrophobic nature of the outer shell on the 3M 1860, 3M 8210, and NS 7210 kept the inoculum on the surface leaving the virus more accessible to UV light exposure. Results between the different matrices were the same at the resolution of the doses with the possible exception of the results for the whole 3M 1860 FFR with virus in simulated lung fluid. Detectable virus remained after a dose of 12·8 J cm^−2^ whereas the UV dose of 13·3 J cm^−2^ (both being 20‐minute exposures) caused inactivation to levels below detection in simulated saliva. Since these doses are not the same it cannot be concluded that there is a significant difference in the inactivation of SARS‐CoV‐2 between these conditions. Additional experiments with greater resolution in UV dose levels are needed to examine this question further. In any case, the similarities were close enough that either simulated saliva or simulated lung fluid can be used to approximate real world background for virus shed from an infected person by sneezing (e.g., saliva) or coughing (simulated lung fluid) with the inactivation results for simulated sunlight expected to be similar. Other considerations worthy of further exploration include varying the virus load placed onto the FFRs or FFR coupons as well as examining the effect of loading virus as an aerosol rather than by droplet inoculation. Such studies would serve to understand virus inactivation by this method more fully and standardize the testing approach.

While this study has shown that FFR decontamination by exposure to light from artificial sun lamps is possible, there are some significant considerations that should be addressed before such an approach is implemented. As was true for the three light sources examined in this study, the intensity of the light will differ between different lamps and fixtures. It is recommended that optical power measurements be made so that exposure times to achieve sufficient doses for inactivation can be estimated. To this end, operators should purchase a radiometer or dosimeter to measure the optical power of their light source and then determine the conditions necessary to achieve the same dose levels. Examples of such devices can be found by searching for UVB digital light meters on the internet with options available for electronic purchase or at home improvement stores. Prior to purchasing, it should be verified that the equipment is capable of measuring UV light in the UVB range and has been calibrated within that range of wavelengths. Furthermore, lamps should be cleaned and changed at the frequency recommended by the manufacturer because dirt and age can reduce the intensity of the light. The last major consideration is that decontamination by exposure to artificial sunlight requires direct line of sight between the lamp and the contaminated surface. Any shadows cast upon the contaminated surface will prevent virus inactivation at those locations. Also, it should be noted that the wavelengths of light with germicidal activity in this artificial sunlight has not been determined. It has been shown that light at 365 nm does not inactivate *B. subtilis* spores (Lin *et al*. 2018) suggesting that lower wavelength light might be more important for virus inactivation, but this has yet to be confirmed. As a result, the spectrum for other artificial sunlight sources should be comparable to those tested here to ensure that the light has germicidal activity. Again, when possible N95 FFRs should be treated as single use items. However, if shortages require N95 FFR reuse, then exposure to artificial sunlight from tanning or horticulture products may be a feasible option. However, further testing to assess variability in devices, illustrate how operators can ensure sufficient UV dosage, and examination of higher inoculum titres which may be present in virus shed from infected persons is needed to increase confidence in this method for actual use. Yet even when decontamination for reuse is being done. It should be noted that guidelines state that FFRs should be discarded if they are soiled or visibly damaged even if a decontamination option is available.

## Conflict of Interest

Battelle does not manufacture or provide N95 FFR decontamination systems that utilize UV light and does not plan to develop or offer such systems in the future. Battelle did manufacture and operate the Battelle Critical Care Decontamination System (CCDS™) which uses vapor phase hydrogen peroxide for decontamination.

## Author Contributions

All authors listed contributed to this work and are entitled to authorship. Dr. Glasbrenner conducted work with TGEV in the BSL‐2 laboratory and prepared the manuscript. Dr. Choi, Ms. Sunderman and Mr. Mladineo conducted work with SARS‐CoV‐2 in the BSL‐3 laboratory. Mr. Richardson and Mr. Keyes conducted the performance testing of the FFRs. Dr. Edwards and Mr. Boyce made the UV test apparatuses and performed the UV dose measurements. Dr. Howard and Dr. Middleton designed the study, analysed the data and assisted with manuscript preparation.

## Supporting information


**Table S1** Simulated saliva composition.
**Table S2**. Simulated lung fluid composition.
**Table S3** Inactivation of TGEV in culture media by the Sperti Fiji Sunlamp on 3M 8511 Coupons.
**Table S4** Inactivation of TGEV in culture media by the Sperti Fiji Sunlamp on NS 7210 Coupons.
**Table S5** Inactivation of TGEV in Culture Media by the Flower Power Sunlamp on 3M 8511 Coupons.
**Table S6** Inactivation of TGEV in culture media by the flower power sunlamp on NS 7210 Coupons.
**Table S7** Inactivation of TGEV in culture media by the tanning bed on 3M 8511 Coupons.
**Table S8** Inactivation of TGEV in culture media by the tanning bed on NS 7210 Coupons.
**Table S9** Inactivation of TGEV in culture media by the tanning bed on 3M 8511 FFRs.
**Table S10** Inactivation of TGEV in culture media by the tanning bed on NS 7210 FFRs.
**Table S11** Inactivation of SARS‐CoV‐2 in culture media by the Sperti Fiji Sunlamp on 3M 8511 Coupons.
**Table S12** Inactivation of SARS‐CoV‐2 in culture media by the Sperti Fiji Sunlamp on NS 7210 Coupons.
**Table S13** Inactivation of SARS‐CoV‐2 in culture media by the Sperti Fiji Sunlamp on 3M 1860 Coupons.
**Table S14** Inactivation of SARS‐CoV‐2 in culture media by the Flower Power Sunlamp on 3M 8511 Coupons.
**Table S15** Inactivation of SARS‐CoV‐2 in culture media by the Flower Power Sunlamp on NS 7210 Coupons.
**Table S16** Inactivation of SARS‐CoV‐2 in culture media by the Flower Power Sunlamp on 3M 1860 Coupons.
**Table S17** Inactivation of SARS‐CoV‐2 in culture media by the tanning bed on 3M 8511 Coupons.
**Table S18** Inactivation of SARS‐CoV‐2 in culture media by the tanning bed on NS 7210 Coupons.
**Table S19** Inactivation of SARS‐CoV‐2 in culture media by the tanning bed on 3M 1860 Coupons.
**Table S20** Inactivation of SARS‐CoV‐2 in culture media by the tanning bed on 3M 8210 Coupons.
**Table S21** Inactivation of SARS‐CoV‐2 in culture media by the tanning bed on 3M 8511 FFRs.
**Table S22** Inactivation of SARS‐CoV‐2 in culture media by the tanning bed on NS 7210 FFRs.
**Table S23** Inactivation of SARS‐CoV‐2 in culture media by the tanning bed on 3M 1860 FFRs.
**Table S24** Inactivation of SARS‐CoV‐2 in culture media by the tanning bed on 3M 8210 FFRs.
**Table S25** Inactivation of SARS‐CoV‐2 in simulated saliva by the tanning bed on FFR Coupons.
