# Supplementary material for: Decontamination of SARS‐CoV‐2 contaminated N95 filtering facepiece respirators using artificial sun lamps
Source: J Appl Microbiol. 2021 Nov 1;131(5):2567–78. doi: 10.1111/jam.15106 (PMC8251224; doi:10.1111/jam.15106)
Supplement: jam15106-sup-0001-TableS1-S25 — Table S1 Simulated saliva composition. Table S2. Simulated lung fluid composition. Table S3 Inactivation of TGEV in culture media by the Sperti Fiji Sunlamp on 3M 8511 Coupons. Table S4 Inactivation of TGEV in culture media by the Sperti Fiji Sunlamp on NS 7210 Coupons. Table S5 Inactivation of TGEV in Culture Media by the Flower Power Sunlamp on 3M 8511 Coupons. Table S6 Inactivation of TGEV in culture media by the flower power sunlamp on NS 7210 Coupons. Table S7 Inactivation of TGEV in culture media by the tanning bed on 3M 8511 Coupons. Table S8 Inactivation of TGEV in culture media by the tanning bed on NS 7210 Coupons. Table S9 Inactivation of TGEV in culture media by the tanning bed on 3M 8511 FFRs. Table S10 Inactivation of TGEV in culture media by the tanning bed on NS 7210 FFRs. Table S11 Inactivation of SARS‐CoV‐2 in culture media by the Sperti Fiji Sunlamp on 3M 8511 Coupons. Table S12 Inactivation of SARS‐CoV‐2 in culture media by the Sperti Fiji Sunlamp on NS 7210 Coupons. Table S13 Inactivation of SARS‐CoV‐2 in culture media by the Sperti Fiji Sunlamp on 3M 1860 Coupons. Table S14 Inactivation of SARS‐CoV‐2 in culture media by the Flower Power Sunlamp on 3M 8511 Coupons. Table S15 Inactivation of SARS‐CoV‐2 in culture media by the Flower Power Sunlamp on NS 7210 Coupons. Table S16 Inactivation of SARS‐CoV‐2 in culture media by the Flower Power Sunlamp on 3M 1860 Coupons. Table S17 Inactivation of SARS‐CoV‐2 in culture media by the tanning bed on 3M 8511 Coupons. Table S18 Inactivation of SARS‐CoV‐2 in culture media by the tanning bed on NS 7210 Coupons. Table S19 Inactivation of SARS‐CoV‐2 in culture media by the tanning bed on 3M 1860 Coupons. Table S20 Inactivation of SARS‐CoV‐2 in culture media by the tanning bed on 3M 8210 Coupons. Table S21 Inactivation of SARS‐CoV‐2 in culture media by the tanning bed on 3M 8511 FFRs. Table S22 Inactivation of SARS‐CoV‐2 in culture media by the tanning bed on NS 7210 FFRs. Table S23 Inactivation of SARS‐CoV‐2 in [file jam15106-sup-0001-tables1-s25.docx]

**Supplemental Information**

Provided in the supplemental information are the components of the simulated saliva and simulated lung fluid solutions along with the tabulated virus inactivation data presented in the results section.

**Matrix Compositions**

***Table SS1. Simulated Saliva Composition.***

| Component | Source | Final Concentration |
| --- | --- | --- |
| MgCl_2_ • 6 H_2_O | Sigma (St. Louis, MO, Cat. No. M2670) | 196.75 μM |
| CaCl_2_ • H_2_O | Sigma (St. Louis, MO, Cat. No. C7902) | 1.17 mM |
| NaHCO_3_ | Fisher (Waltham, MA, Cat. No. S233) | 5.00 mM |
| KH_2_PO_4_ | Fisher (Waltham, MA, Cat. No. S233) | 15.4 mM |
| K_2_HPO_4_ | MP Biomedicals (Santa Ana, CA, Cat. No. 151945) | 24.6 mM |
| NH_4_Cl | Sigma (St. Louis, MO, Cat. No. 254134) | 2.06 mM |
| KSCN | Sigma (St. Louis, MO, Cat. No. 207799) | 1.96 mM |
| (NH_2_)_2_CO | Sigma (St. Louis, MO, Cat. No. U5378) | 2.00 mM |
| NaCl | Sigma (St. Louis, MO, Cat. No. S6191) | 15.06 mM |
| KCl | Sigma (St. Louis, MO, Cat. No. P3911) | 13.96 mM |
| Mucin (porcine gastric type III) | Lee Biosoultions (Maryland Heights, MO, Cat. No. M1778) | 3 g/L |
| ddH_2_O |  | To volume |

***Table S2. Simulated Lung Fluid Composition.***

| Component | Source | Final Concentration |
| --- | --- | --- |
| DPPG | Avanti (Alabaster, AL, Cat. No. 840455P) | 0.5 mg/mL |
| DPPC | Avanti (Alabaster, AL, Cat. No. 850355P) | 4.8 mg/mL |
| Cholesterol | Sigma (St. Louis, MO, Cat. No. C4045) | 0.1 mg/mL |
| Albumin | Sigma (St. Louis, MO, Cat. No. A3782) | 8.8 mg/mL |
| IgG | Sigma (St. Louis, MO, Cat. No. I4506) | 2.6 mg/mL |
| Transferrin | Sigma (St. Louis, MO, Cat. No. T8158) | 1.5 mg/mL |
| L-Ascorbic acid | Sigma (St. Louis, MO, Cat. No. A5960) | 122 μM |
| Uric acid | Sigma (St. Louis, MO, Cat. No. U0881) | 95 μM |
| Glutathione | Sigma (St. Louis, MO, Cat. No. PHR1359) | 161 μM |
| Gentamicin | Sigma (St. Louis, MO, Cat. No. G1397) | 0.05 mg/mL |
| 1x HBSS | Gibco (Carlsbad, CA, Cat. No. 14025-076) | 10.04 mL |

Tabulated inactivation data

***Table S3. Inactivation of TGEV in Culture Media by the Sperti Fiji Sunlamp on 3M 8511 Coupons***

| Time (min) | UV Dose_300-400nm_ (J/cm^2^) | Log_10_ (TCID_50_) ± St. Dev. |
| --- | --- | --- |
| 0 | 0.0 | 3.41 ± 0.15 |
| 0.7 | 0.071 | 3.12 ± 0.55 |
| 7 | 0.75 | 3.45 ± 0.31 |
| 77 | 6.83 | BD |

BD – Below Detection

***Table S4. Inactivation of TGEV in Culture Media by the Sperti Fiji Sunlamp on NS 7210 Coupons***

| Time (min) | UV Dose_300-400nm_ (J/cm^2^) | Log_10_ (TCID_50_) ± St. Dev. |
| --- | --- | --- |
| 0 | 0.0 | 3.73 ± 0.71 |
| 0.7 | 0.074 | 2.31 ± 0.75 |
| 7 | 0.75 | 2.81 ± 1.28 |
| 77 | 8.00 | BD |

BD – Below Detection

***Table S5. Inactivation of TGEV in Culture Media by the Flower Power Sunlamp on 3M 8511 Coupons***

| Time (min) | UV Dose_300-400nm_ (J/cm^2^) | Log_10_ (TCID_50_) ± St. Dev. |
| --- | --- | --- |
| 0 | 0.0 | 3.42 ± 0.58 |
| 3.5 | 0.051 | 2.67 ± 0.06 |
| 35 | 0.99 | BD |
| 70 | 2.02 | BD |

BD – Below Detection

***Table S6. Inactivation of TGEV in Culture Media by the Flower Power Sunlamp on NS 7210 Coupons***

| Time (min) | UV Dose_300-400nm_ (J/cm^2^) | Log_10_ (TCID_50_) ± St. Dev. |
| --- | --- | --- |
| 0 | 0.0 | 4.04 ± 0.58 |
| 3.5 | 0.051 | 2.50 ± 0.46 |
| 35 | 1.03 | BD |
| 70 | 2.02 | BD |

BD – Below Detection

***Table S7. Inactivation of TGEV in Culture Media by the Tanning Bed on 3M 8511 Coupons***

| Time (min) | UV Dose_300-400nm_ (J/cm^2^) | Log_10_ (TCID_50_) ± St. Dev. |
| --- | --- | --- |
| 0 | 0.0 | 4.00 ± 0.84 |
| 0.22 | 0.14 | 3.20 ± 0.33 |
| 0.43 | 0.27 | 2.75 ± 0.22 |
| 0.67 | 0.44 | 3.14 ± 0.42 |
| 2 | 1.32 | 2.54 ± 0.05 |
| 5 | 3.60 | BD |

BD – Below Detection

***Table S8. Inactivation of TGEV in Culture Media by the Tanning Bed on NS 7210 Coupons***

| Time (min) | UV Dose_300-400nm_ (J/cm^2^) | Log_10_ (TCID_50_) ± St. Dev. |
| --- | --- | --- |
| 0 | 0.0 | 4.51 ± 0.71 |
| 0.22 | 0.14 | 3.58 ± 0.11 |
| 0.43 | 0.27 | 2.82 ± 0.73 |
| 0.67 | 0.42 | 1.97 ± 0.42 |
| 2 | 1.31 | BD |

BD – Below Detection

***Table S9. Inactivation of TGEV in Culture Media by the Tanning Bed on 3M 8511 FFRs***

| Time (min) | UV Dose_300-400nm_ (J/cm^2^) | Log_10_ (TCID_50_) ± St. Dev. |
| --- | --- | --- |
| 0 | 0.0 | 3.83 ± 0.77 |
| 0.22 | 0.12 | 3.76 ± 0.36 |
| 0.43 | 0.25 | 3.31 ± 0.31 |
| 0.67 | 0.38 | 3.51 ± 0.12 |
| 2 | 1.19 | 3.19 ± 0.33 |

BD – Below Detection

***Table S10. Inactivation of TGEV in Culture Media by the Tanning Bed on NS 7210 FFRs***

| Time (min) | UV Dose_300-400nm_ (J/cm^2^) | Log_10_ (TCID_50_) ± St. Dev. |
| --- | --- | --- |
| 0 | 0.0 | 3.89 ± 0.60 |
| 0.22 | 0.14 | 3.35 ± 0.52 |
| 0.43 | 0.27 | 3.08 ± 0.14 |
| 0.67 | 0.42 | 2.78 ± 0.58 |
| 2 | 1.31 | 1.00 ± 0.44 |

BD – Below Detection

***Table S11. Inactivation of*** ***SARS-CoV-2 in Culture Media by the Sperti Fiji Sunlamp on 3M 8511 Coupons***

| Time (min) | UV Dose_300-400nm_ (J/cm^2^) | Log_10_ (TCID_50_) ± St. Dev. |
| --- | --- | --- |
| 0 | 0.0 | 3.28 ± 0.77 |
| 1 | 0.084 | 3.33 ± 0.37 |
| 9 | 0.76 | 1.81 ± 0.71 |
| 45 | 3.80 | BD |

BD – Below Detection

***Table S12. Inactivation of SARS-CoV-2 in Culture Media by the Sperti Fiji Sunlamp on NS 7210 Coupons***

| Time (min) | UV Dose_300-400nm_ (J/cm^2^) | Log_10_ (TCID_50_) ± St. Dev. |
| --- | --- | --- |
| 0 | 0.0 | 4.45 ± 0.0 |
| 1 | 0.11 | 2.18 ± 0.75 |
| 9 | 1.02 | 1.50 ± 0.47 |
| 45 | 5.10 | BD |

BD – Below Detection

***Table S13. Inactivation of SARS-CoV-2 in Culture Media by the Sperti Fiji Sunlamp on 3M 1860 Coupons***

| Time (min) | UV Dose_300-400nm_ (J/cm^2^) | Log_10_ (TCID_50_) ± St. Dev. |
| --- | --- | --- |
| 0 | 0.0 | 4.17 ± 0.17 |
| 1 | 0.092700 | 2.86 ± 0.21 |
| 9 | 0.834300 | BD |
| 45 | 4.171500 | BD |

BD – Below Detection

***Table S14. Inactivation of SARS-CoV-2 in Culture Media by the Flower Power Sunlamp on 3M 8511 Coupons***

| Time (min) | UV Dose_300-400nm_ (J/cm^2^) | Log_10_ (TCID_50_) ± St. Dev. |
| --- | --- | --- |
| 0 | 0.0 | 4.45 ± 0.0 |
| 1 | 0.055 | 3.75 ± 0.37 |
| 9 | 0.49 | 2.93 ± 1.7 |
| 45 | 2.46 | 3.10 ± 0.32 |

BD – Below Detection

***Table S15. Inactivation of SARS-CoV-2 in Culture Media by the Flower Power Sunlamp on NS 7210 Coupons***

| Time (min) | UV Dose_300-400nm_ (J/cm^2^) | Log_10_ (TCID_50_) ± St. Dev. |
| --- | --- | --- |
| 0 | 0.0 | 5.01 ± 0.0 |
| 1 | 0.056 | 3.66 ± 0.21 |
| 9 | 0.50 | BD |
| 45 | 2.45 | BD |

BD – Below Detection

***Table S16. Inactivation of SARS-CoV-2 in Culture Media by the Flower Power Sunlamp on 3M 1860 Coupons***

| Time (min) | UV Dose_300-400nm_ (J/cm^2^) | Log_10_ (TCID_50_) ± St. Dev. |
| --- | --- | --- |
| 0 | 0.0 | 5.01 ± 0.0 |
| 1 | 0.05546667 | 3.84 ± 0.21 |
| 9 | 0.499200 | BD |
| 45 | 2.442667 | BD |

BD – Below Detection

***Table S17. Inactivation of SARS-CoV-2 in Culture Media by the Tanning Bed on 3M 8511 Coupons***

| Time (min) | UV Dose_300-400nm_ (J/cm^2^) | Log_10_ (TCID_50_) ± St. Dev. |
| --- | --- | --- |
| 0 | 0.0 | 3.84 ± 0.081 |
| 4 | 2.59 | 2.35 ± 0.14 |
| 8 | 5.13 | 1.93 ± 0.14 |
| 14 | 8.60 | 1.88 ± 0.081 |
| 30 | 15.6 | BD |

BD – Below Detection

***Table S18. Inactivation of SARS-CoV-2 in Culture Media by the Tanning Bed on NS 7210 Coupons***

| Time (min) | UV Dose_300-400nm_ (J/cm^2^) | Log_10_ (TCID_50_) ± St. Dev. |
| --- | --- | --- |
| 0 | 0.0 | 5.01 ± 0.0 |
| 0.5 | 0.32 | 3.56 ± 0.081 |
| 1.5 | 0.84 | 2.82 ± 0.16 |
| 5 | 2.91 | 1.19 ± 1.0 |
| 20 | 12.4 | BD |

BD – Below Detection

***Table S19. Inactivation of SARS-CoV-2 in Culture Media by the Tanning Bed on 3M 1860 Coupons***

| Time (min) | UV Dose_300-400nm_ (J/cm^2^) | Log_10_ (TCID_50_) ± St. Dev. |
| --- | --- | --- |
| 0 | 0.0 | 5.01 ± 0.0 |
| 2 | 1.29 | 2.02 ± 0.16 |
| 4 | 2.60 | 2.44 ± 0.08 |
| 10 | 6.50 | BD |

BD – Below Detection

***Table S20. Inactivation of SARS-CoV-2 in Culture Media by the Tanning Bed on 3M 8210 Coupons***

| Time (min) | UV Dose_300-400nm_ (J/cm^2^) | Log_10_ (TCID_50_) ± St. Dev. |
| --- | --- | --- |
| 0 | 0.0 | 3.42 ± 0.16 |
| 2 | 1.14 | 3.05 ± 0.14 |
| 4 | 2.59 | 2.26 ± 0.29 |
| 12 | 6.99 | 1.84 ± 0.08 |

BD – Below Detection

***Table S21. Inactivation of SARS-CoV-2 in Culture Media by the Tanning Bed on 3M 8511*** ***FFRs***

| Time (min) | UV Dose_300-400nm_ (J/cm^2^) | Log_10_ (TCID_50_) ± St. Dev. |
| --- | --- | --- |
| 0 | 0.0 | 4.27 ± 0.31 |
| 4 | 2.59 | 2.91 ± 0.30 |
| 8 | 5.15 | 2.73 ± 0.13 |
| 14 | 9.08 | BD |

BD – Below Detection

***Table S22. Inactivation of SARS-CoV-2 in Culture Media by the Tanning Bed on NS 7210 FFRs***

| Time (min) | UV Dose_300-400nm_ (J/cm^2^) | Log_10_ (TCID_50_) ± St. Dev. |
| --- | --- | --- |
| 0 | 0.0 | 4.62 ± 0.070 |
| 2 | 1.16 | 4.55 ± 0.35 |
| 4 | 2.09 | 3.96 ± 0.40 |
| 12 | 7.15 | BD |

BD – Below Detection

***Table S23. Inactivation of SARS-CoV-2 in Culture Media by the Tanning Bed on 3M 1860 FFRs***

| Time (min) | UV Dose_300-400nm_ (J/cm^2^) | Log_10_ (TCID_50_) ± St. Dev. |
| --- | --- | --- |
| 0 | 0.0 | 4.62 ± 0.35 |
| 2 | 1.15 | 3.78 ± 0.13 |
| 4 | 2.09 | 3.12 ± 0.65 |
| 10 | 5.75 | BD |

BD – Below Detection

***Table S24. Inactivation of SARS-CoV-2 in Culture Media by the Tanning Bed on 3M 8210 FFRs***

| Time (min) | UV Dose_300-400nm_ (J/cm^2^) | Log_10_ (TCID_50_) ± St. Dev. |
| --- | --- | --- |
| 0 | 0.0 | 4.69 ± 0.26 |
| 0.5 | 0.32 | 3.92 ± 0.40 |
| 1.5 | 0.88 | 3.68 ± 0.14 |
| 5 | 3.22 | 2.16 ± 0.78 |
| 20 | 13.2 | BD |

BD – Below Detection

***Table S25. Inactivation of SARS-CoV-2 in Simulated Saliva by the Tanning Bed on FFR Coupons***

| FFR Type | UV Dose_300-400nm_ (J/cm^2^) (Time in Minutes) | Log_10_ (TCID_50_) ± St. Dev. |
| --- | --- | --- |
| 3M 8511 | 0.0 (0.0) | 4.17 ± 0.0 |
|  | 39.8 (60) | BD |
| NS 7210 | 0.0 (0.0) | 4.45 ± 0.24 |
|  | 13.8 (20) | BD |
| 3M 1860 | 0.0 (0.0) | 4.50 ± 0.16 |
|  | 13.8 (20) | BD |

BD – Below Detection

***Table S25. Inactivation of SARS-CoV-2 in Simulated Lung Fluid by the Tanning Bed on FFR Coupons***

| FFR Type | UV Dose_300-400nm_ (J/cm^2^) (Time in Minutes) | Log_10_ (TCID_50_) ± St. Dev. |
| --- | --- | --- |
| 3M 8511 | 0.0 (0.0) | 5.70 ± 0.14 |
|  | 39.8 (60) | BD |
| NS 7210 | 0.0 (0.0) | 5.56 ± 0.24 |
|  | 13.8 (20) | BD |
| 3M 1860 | 0.0 (0.0) | 5.67 ± 0.080 |
|  | 13.8 (20) | 3.05 ± 0.14 |
|  | 26.5 (40) | BD |

BD – Below Detection
